# Polyphenols and Their Impact on Human Health

**DOI:** 10.3390/ijms242316683

**Published:** 2023-11-24

**Authors:** Lucia Panzella

**Affiliations:** Department of Chemical Sciences, University of Naples “Federico II”, Via Cintia 4, I-80126 Naples, Italy; panzella@unina.it

Given their potent antioxidant and biological properties [1,2,3], and in view of their widespread occurrence not only in foods, but also in easily accessible sources such as waste materials from agri-food industries [1,4,5,6], polyphenols have attracted increasing attention with regard to their possible use as food supplements and as functional ingredients for biomedical and cosmetic applications [7,8,9,10].

In this context, the Special Issue “Polyphenols and Their Impact on Human Health” aimed to collect articles reporting innovative applications of natural polyphenols from edible or non-edible sources in the field of nutrition and biomedicine, spanning a range of topics from food supplements or functional foods that prevent oxidative-stress-related diseases to additives in biomedical devices, skincare formulations and cosmetic products. Eight contributions (five research articles and three reviews) have been published in this Special Issue, as listed below:

1. Gravina, C.; Formato, M.; Piccolella, S.; Fiorentino, M.; Stinca, A.; Pacifico, S.; Esposito, A. Lavandula austroapennina (Lamiaceae): Getting Insights into Bioactive Polyphenols of a Rare Italian Endemic Vascular Plant. *Int. J. Mol. Sci.* **2023**, *24*, 8038. https://doi.org/10.3390/ijms24098038
2. Song, H.; Kang, S.; Yu, Y.; Jung, S.Y.; Park, K.; Kim, S.-M.; Kim, H.-J.; Kim, J.G.; Kim, S.E. In Vitro Anti-Inflammatory and Antioxidant Activities of pH-Responsive Resveratrol-Urocanic Acid Nano-Assemblies. *Int. J. Mol. Sci.* **2023**, *24*, 3843. https://doi.org/10.3390/ijms24043843
3. Sánchez-Medina, A.; Redondo-Puente, M.; Dupak, R.; Bravo-Clemente, L.; Goya, L.; Sarriá, B. Colonic Coffee Phenols Metabolites, Dihydrocaffeic, Dihydroferulic, and Hydroxyhippuric Acids Protect Hepatic Cells from TNF-α-Induced Inflammation and Oxidative Stress. *Int. J. Mol. Sci.* **2023**, *24*, 1440. https://doi.org/10.3390/ijms24021440
4. Rahn, C.; Bakuradze, T.; Stegmüller, S.; Galan, J.; Niesen, S.; Winterhalter, P.; Richling, E. Polyphenol-Rich Beverage Consumption Affecting Parameters of the Lipid Metabolism in Healthy Subjects. *Int. J. Mol. Sci.* **2023**, *24*, 841. https://doi.org/10.3390/ijms24010841
5. Laghezza Masci, V.; Bernini, R.; Villanova, N.; Clemente, M.; Cicaloni, V.; Tinti, L.; Salvini, L.; Taddei, A.R.; Tiezzi, A.; Ovidi, E. In Vitro Anti-Proliferative and Apoptotic Effects of Hydroxytyrosyl Oleate on SH-SY5Y Human Neuroblastoma Cells. *Int. J. Mol. Sci.* **2022**, *23*, 12348. https://doi.org/10.3390/ijms232012348
6. Lippolis, T.; Cofano, M.; Caponio, G.R.; De Nunzio, V.; Notarnicola, M. Bioaccessibility and Bioavailability of Diet Polyphenols and Their Modulation of Gut Microbiota. *Int. J. Mol. Sci.* **2023**, *24*, 3813. https://doi.org/10.3390/ijms24043813
7. Maisto, M.; Iannuzzo, F.; Novellino, E.; Schiano, E.; Piccolo, V.; Tenore, G.C. Natural Polyphenols for Prevention and Treatment of Urinary Tract Infections. *Int. J. Mol. Sci.* **2023**, *24*, 3277. https://doi.org/10.3390/ijms24043277
8. Aleksandrova, S.; Alexova, R.; Dragomanova, S.; Kalfin, R.; Nicoletti, F.; Fagone, P.; Petralia, M.C.; Mangano, K.; Tancheva, L. Preventive and Therapeutic Effects of Punica granatum L. Polyphenols in Neurological Conditions. *Int. J. Mol. Sci.* **2023**, *24*, 1856. https://doi.org/10.3390/ijms24031856

Contribution 1 reported preliminary data on the antioxidant and bioactivity, in terms of wound closure ability, of polyphenol-rich extracts from several organs of *Lavandula austroapennina*. Extracts rich in hydroxycinnamoyl derivatives were found to be particularly effective as antioxidants in chemical assays compared to flavonoid-rich extracts, which induced rapid wound closure on HaCaT cells even at low concentrations (1 μg/mL).

Contribution 2 described the preparation, as well as the antioxidant and anti-inflammatory properties, of pH-responsive nanoparticles composed of resveratrol and urocanic acid.

Contribution 3 reported the hepatoprotective effects against inflammation and oxidative stress of the main metabolites of hydroxycinnamic acids; that is, dihydrocaffeic, dihydroferulic, and hydroxyhippuric acids, at concentrations (0.5–10 μM) that are likely to be found in biological compartments after coffee ingestion.

Contribution 4 demonstrated the beneficial effects of a polyphenol-rich beverage containing anthocyanins, flavonoids, chlorogenic acids and ellagitannins with regard to lipid metabolism and DNA integrity in a placebo-controlled human intervention study.

Contribution 5 described hydroxytyrosyl oleate as a promising derivative of the natural phenol hydroxytyrosol, that is endowed with a higher cellular bioavailability. The synthesized compound maintained the anti-proliferative and apoptotic effects of the parent compound at lower concentrations in human neuroblastoma cells.

Contribution 6 explores the interaction of dietary polyphenols, with particular reference to gallic acid, resveratrol, flavonoids and proanthocyanidins, with gut microbiota as an important aspect to be considered when evaluating the health beneficial effects of these compounds. Microencapsulation strategies for improving the positive modulation of gut microbiota induced by polyphenols are also discussed.

Contribution 7 reports the results of in vitro and in vivo studies demonstrating the potential therapeutic efficacy and the mechanism of action of dietary polyphenols (proanthocyanidins, catechins, resveratrol, caffeic acid, and quercetin) against urinary tract infections.

Finally, in contribution 8, the preventive and therapeutic effects, as well as the mechanism of action of phenolic compounds from *Punica granatum* L., mainly ellagitannins, in neurological conditions are reviewed.

The authors of these contributions originate not only from Italy and Spain—countries that are traditionally involved in the scientific research on phenolic compounds, as these compounds are largely responsible for the beneficial effects of the Mediterrean diet—but also from Korea, Germany, Bulgaria, and Slovakia. In addition, the high diversity in the content and scientific approaches presented clearly demonstrates how broad the research field on polyphenols is, and this is even more true considering the topic of the Special Issue was limited to the impact of these compounds on human health.

A fundamental observation emerging from the published papers is that although natural phenolic compounds are intrinsically a source of chemical multifunctionality and may offer unrivalled opportunities as eco-friendly and sustainable compounds, a deep understanding of the structure–property relationships hidden by the complexity of these compounds is crucial to the development of rational strategies for their full exploitation as functional ingredients in the health, food and/or cosmetic sectors.

The collected contributions also help to define the research priorities and underexplored aspects in this field, ranging from the re-evaluation of local, small-scale agricultural products as underexplored sources of polyphenols, the exploration of novel potential applications, and the design of proper formulations or chemical modifications to enhance the biological activity and bioavailability of these compounds, to the evaluation of the role of dietary polyphenol metabolites in the healthy effects of these compounds and the development of in vivo human studies involving not only an extended number but also specific categories of volunteers to develop, for example, new dietary treatments and to obtain more detailed information on the impact of polyphenols on human health.

## References

[1] Jimenez-Lopez C., Fraga-Corral M., Carpena M., García-Oliveira P., Echave J., Pereira A.G., Lourenço-Lopes C., Prieto M.A., Simal-Gandara J. (2020). Agriculture waste valorisation as a source of antioxidant phenolic compounds within a circular and sustainable bioeconomy. Food Funct..

[2] Cory H., Passarelli S., Szeto J., Tamez M., Mattei J. (2018). The role of polyphenols in human health and food systems: A mini-review. Front. Nutr..

[3] Rahman M.M., Rahaman M.S., Islam M.R., Rahman F., Mithi F.M., Alqahtani T., Almikhlafi M.A., Alghamdi S.Q., Alruwaili A.S., Hossain M.S. (2022). Role of phenolic compounds in human disease: Current knowledge and future prospects. Molecules.

[4] Gullón B., Yáñez R. (2022). Recovery of high value-added compounds from food by-product. Foods.

[5] Panzella L., Moccia F., Nasti R., Marzorati S., Verotta L., Napolitano A. (2020). bioactive phenolic compounds from agri-food wastes: An update on green and sustainable extraction methodologies. Front. Nutr..

[6] Moccia F., Agustin-Salazar S., Verotta L., Caneva E., Giovando S., D’Errico G., Panzella L., d’Ischia M., Napolitano A. (2020). Antioxidant properties of agri-food byproducts and specific boosting effects of hydrolytic treatments. Antioxidants.

[7] Rahim M.A., Kristufek S.L., Pan S., Richardson J.J., Caruso F. (2019). Phenolic building blocks for the assembly of functional materials. Angew. Chem. Int. Ed..

[8] Caleja C., Ribeiro A., Barreiro M.F., Ferreira I.C.F.R. (2017). Phenolic compounds as nutraceuticals or functional food ingredients. Curr. Pharm. Des..

[9] Panzella L., Napolitano A. (2019). Natural and bioinspired phenolic compounds as tyrosinase inhibitors for the treatment of skin hyperpigmentation: Recent advances. Cosmetics.

[10] Albuquerque B.R., Heleno S.A., Oliveira M.B.P.P., Barros L., Ferreira I.C.F.R. (2021). Phenolic compounds: Current industrial applications, limitations and future challenges. Food Funct..

